# A new method to evaluate temperature vs. pH activity profiles for biotechnological relevant enzymes

**DOI:** 10.1186/s13068-017-0923-9

**Published:** 2017-10-11

**Authors:** J. Herlet, P. Kornberger, B. Roessler, J. Glanz, W. H. Schwarz, W. Liebl, V. V. Zverlov

**Affiliations:** 1 Department of Microbiology, TUM School of Life Sciences Weihenstephan, Technical University of Munich, Emil-Ramann-Str. 4, 85354 Freising-Weihenstephan, Germany; 2 Institute of Molecular Genetics, Russian Academy of Science, Kurchatov Sq. 2, 123182 Moscow, Russia

**Keywords:** Glycoside hydrolase, pH optimum, Temperature optimum, Contour plot, Enzyme activity, Celluclast^®^, Cel8A, Endoglucanase, Cellulase

## Abstract

**Background:**

Glycoside hydrolases are important for various industrial and scientific applications. Determination of their temperature as well as pH optima and range is crucial to evaluate whether an enzyme is suitable for application in a biotechnological process. These basic characteristics of enzymes are generally determined by two separate measurements. However, these lead to a two-dimensional assessment of the pH range at one temperature (and vice versa) and do not allow prediction of the relative enzymatic performance at any pH/temperature combination of interest. In this work, we demonstrate a new method that is based on experimental data and visualizes the relationship among pH, temperature, and activity at a glance in a three-dimensional contour plot.

**Results:**

In this study, we present a method to determine the relative activity of an enzyme at 96 different combinations of pH and temperature in parallel. For this purpose, we used a gradient PCR cycler and a citrate–phosphate-based buffer system in microtiter plates. The approach was successfully tested with various substrates and diverse assays for glycoside hydrolases. Furthermore, its applicability was demonstrated for single enzymes using the endoglucanase Cel8A from *Clostridium thermocellum* as well as the commercially available complex enzyme mixture Celluclast^®^. Thereby, we developed a fast and adaptable method to determine simultaneously both pH and temperature ranges of enzymes over a wide range of conditions, an easy transformation of the experimental data into a contour plot for visualization, and the necessary controls. With our method, the suitability of an enzyme or enzyme mixture for any chosen combination of temperature and pH can easily be assessed at a glance.

**Conclusions:**

We propose a method that offers significant advantages over commonly used methods to determine the pH and temperature ranges of enzymes. The overall relationship among pH, temperature, and activity is visualized. Our method could be applied to evaluate exactly what conditions have to be met for optimal utilization of an enzyme or enzyme mixture for both lab-scale and industrial processes. Adaptation to other enzymes, including proteases, should be possible and the method may also lead to a platform for additional applications, such as inactivation kinetics analysis.

**Electronic supplementary material:**

The online version of this article (doi:10.1186/s13068-017-0923-9) contains supplementary material, which is available to authorized users.

## Background

Hydrolases (E.C. 3) are enzymes that can cleave different bonds such as ester, peptide, or glycoside bonds. Among many other applications, these enzymes play an important role in the conversion of biomass to fermentable sugars. These sugars can be used for the production of bioethanol and other bio-based chemicals as well as polymers [1, 2]. Enzymatic degradation of biomass can be performed under mild conditions with a low environmental impact and is, therefore, preferred over thermo-chemical approaches. However, degradation of the complex components of lignocellulosic biomass requires the use of multiple enzymes [3, 4], preferably in one reaction mixture. As a consequence, all enzymes within this one-pot reaction have to be compatible both with each other and with the employed process conditions, such as the temperature and pH. Applying the most suitable pH and temperature conditions for all enzymes can lead to a reduction in the amount of enzyme needed in a one-pot reaction, which is crucial for the feasibility of enzymatic bioconversions [5–7].

Conventionally, enzyme characteristics in regard to temperature and pH are determined by measuring both parameters separately. This two-dimensional approach, however, treats temperature and pH as two independent variables. This method, while allowing a quick assessment, is very limited in the information obtained. Any data about the enzyme’s activity that was not directly measured cannot be derived. It is, therefore, not possible to assess accurately what conditions are suitable to use, for example, for two enzymes simultaneously. A range for the optima can be stated, but this is based on the assumption that the correlations between temperature and activity on the one hand as well as between pH and activity on the other hand are independent of one another.

Statistical experimental design approaches, which depict the effects of temperature and pH on the enzyme’s activity by a response surface analysis, are more advanced than the conventional approach. Most studies use a Box–Wilson central composite design with an embedded factorial design. The experimental setup of these studies usually consists of 11 runs, which correspond to four cube points, four axial points, and three central points [8–10]. The effects of temperature and pH on the enzyme’s activity can then be visualized using a response surface map, which is derived from statistical calculation by a mathematical tool. Hence, it does not show experimental data and again has to be tested to determine its accuracy by additional experiments. The use of a statistical design does reduce the number of samples, especially when the effects of several variables are investigated simultaneously. However, in the case of pH and temperature optimum determination, only the effects of two variables (pH and temperature) on one factor (activity) are determined.

In this study, we developed a method to determine the effects of pH and temperature on an enzyme’s activity simultaneously using an experimental approach rather than a statistical one. A thermocycler with gradient function and 96-well PCR plates are used to compare the impact of 96 different combinations of pH and temperature on the enzyme’s activity simultaneously. The data set is then converted into a contour plot for easy visualization. We show that our method works with a range of different hydrolase assays, such as 3,5-dinitrosalicylic acid (DNSA) assay [11, 12], Azo-CM-cellulose dye liberation assay (Megazyme), an assay measuring the release of nitrophenol from *para*-nitrophenol (*p*-NP) glycosides, as well as the d-Glucose HK Assay (Megazyme), using a straw-based natural substrate. The method was evaluated using the endoglucanase Cel8A, a well-characterized cellulase from *Clostridium thermocellum* and the commercially available cellulose-degrading enzyme mixture Celluclast^®^ (Novozymes). Our method provides an easy, cheap, and fast in-depth characterization of the combined effects of temperature and pH on an enzyme’s relative activity. The range in which an enzyme or enzyme mixture is most suitable for application can be described accurately and visualized for at-a-glance assessment. To verify the data, appropriate controls have also been developed.

## Methods

### Buffer

A citrate–phosphate buffer system was used for all assays due to its pH buffering capacity within the pH range of 4–8, which is largely unaffected by temperature. Two solutions were used to prepare the buffers at room temperature: solution A consisted of 0.2 M citric acid with 0.1 M NaCl and solution B consisted of 0.4 M disodium hydrogen phosphate with 0.1 M NaCl. All cavities of a Riplate^®^ 2 ml 96-deepwell plate from Carl Roth (Karlsruhe, Germany) were filled with 1.5 ml of buffer (row A: pH 4.0, row B: pH 4.6, row C: pH 5.2, row D: pH 5.8, row E: pH 6.4, row F: pH 7.0, row G: pH 7.6, row H: pH 8.0) to obtain a buffer reservoir that can be used to perform several assays. To test the temperature dependency of the buffers, each one was gradually heated to 80 °C while recording the pH (taking into account the temperature dependency of the pH electrode).

### Enzymes and substrates

Cel8A from *C. thermocellum* was produced using the pET-24c(+) expression plasmid (Thermo Fisher Scientific, Waltham, MA, USA) in *E.* *coli* BL21Star™ cells (Thermo Fisher Scientific), as described by Mechelke et al. [13]. Celluclast^®^ 1.5 l was purchased from Sigma Aldrich (St. Louis, MO, USA). Barley-β-glucan (BBG, medium viscosity) and arabinoxylan (AX, wheat, medium viscosity) were obtained from Megazyme (Bray, Ireland). *para*-Nitrophenyl-β-d-glucopyranoside (*p*-NP-β-d-glucopyranoside) was purchased from Sigma Aldrich. Azo-CM-cellulose was obtained from Megazyme. Untreated wheat straw was sterilized in a Systec VX-150 autoclave (Linden, Germany) at 121 °C for 20 min with 1% NaOH (v/v), washed with water until it reached a neutral pH, and dried at 60 °C. The dried straw was pulverized using the planetary mono mill PULVERISETTE 6 and sieved using a ANALYSETTE 3 SPARTAN sieve shaker, both from Fritsch (Einersheim, Germany). The sieved fraction with a particle size of less than 50 µm was used as a substrate.

To test whether the substrates were stable within the respective assay conditions (pH, temperature, and time), all substrates were incubated according to our standard contour plot protocol for the respective assay with water instead of enzyme. The average background of each substrate was used as a blank for the calculation of the contour plot.

In accordance with the conventional determination of pH and temperature optima, a dilution series of each enzyme or enzyme mix was produced to determine the adequate amount of enzyme deployed in the assay (data not shown). The final concentrations for Cel8A and Celluclast^®^ within the different assays and for the diverse substrates were as follows: Cel8A (BBG, DNSA assay): 0.5 µg/ml, Cel8A (Azo-CM-cellulose, Azo-CM-cellulose assay): 5 µg/ml, Celluclast^®^ (AX, DNSA assay): 200 µg/ml, Celluclast^®^ (barley-β-glucan, DNSA assay): 5 µg/ml, Celluclast^®^ (*p*-NP-β-d-glucopyranoside, *p*-NP assay): 50 µg/ml, and Celluclast^®^ (straw-based substrate, d-glucose HK assay): 250 µg/ml.

### Experimental procedure

#### DNSA assay

The DNSA assay solution was prepared according to Miller [12]. PrimaPLATE 96-well PCR plates from Steinbrenner (Wiesenbach, Germany) were used for the reactions. All steps except for the addition of the enzyme dilution were performed using an Integra (Biebertal, Germany) Viaflo 96-channel electronic pipette. The addition of the enzyme dilution was performed using an Integra Voyager 8-channel electronic pipette to minimize the loss of enzyme caused by the reservoir. For the DNSA assay, 50 µl of the respective buffer solution was added to each well from a 96-deepwell plate (see buffer section) into a PCR plate. Next, 50 µl of a 1% (w/v) substrate solution (arabinoxylan or barley-β-glucan) was added. As the last step, 50 µl of the enzyme dilution was added and the solution was mixed thoroughly. The PCR plate was sealed using a Heatsealer 4S (Steinbrenner) set to 2.5 s at 175 °C. Subsequently, the plate was incubated in a Primus96 advanced PCR cycler from PEQLAB (Erlangen, Germany) for 90 min using the gradient mode with a ± 19.9 °C span. The gradient for Cel8A was set to 60.1 ± 19.9 °C (resulting in 40.2, 43.9, 47.5, 51.1, 54.7, 58.3, 61.9, 65.5, 69.1, 72.7, 76.3, and 80.0 °C for columns 1 to 12, respectively), while the gradient for Celluclast^®^ was set to 55 ± 19.9 °C (resulting in 35.1, 38.8, 42.4, 46.0, 49.6, 53.2, 56.8, 60.4, 64.0, 67.6, 71.2, and 74.9 °C). The reaction was stopped by transferring the plate into ice water. Fifty microliters from each well were pipetted into a new PCR plate and subsequently 75 µl of DNSA solution was added and the solution was mixed thoroughly. The plate was sealed once again and incubated at 95 °C for 5 min using a PCR cycler. The reaction was stopped by transferring the plate into ice water. 75 µl from each well was transferred into a Carl Roth Rotilabo^®^ 96-well F-bottom plate and the absorption at 540 nm (A_540_) was determined using a Sunrise™ microplate reader from Tecan (Männedorf, Switzerland).

#### Azo-CM-cellulose assay

Two grams of Azo-CM-cellulose were added to 80 ml of boiling MilliQ-water under vigorous stirring until the substrate had completely dissolved. The precipitation buffer was prepared in accordance with the manufacturer’s manual (Megazyme). A total of 13.5 µl of citrate–phosphate buffer from the 96-deepwell plate (see buffer section) was added into a PCR plate. Subsequently, 20 µl of substrate solution and finally 6.5 µl of enzyme dilution were added and the solution was mixed thoroughly. The plate was then sealed and incubated as described for the DNSA assay. After cooling down the plate on ice water, 110 µl of precipitation buffer was added and the solution was mixed thoroughly. The plate was sealed again and centrifuged at 1600×*g* for 10 min. The centrifuge brake was set to low to avoid loosening of the pellet. Then, 100 µl of the supernatant was transferred into a 96-well F-bottom plate and the absorption at 595 nm (A_595_) was determined as mentioned above.

#### *p*-NP assay

*p*-NP substrates were dissolved in dimethylformamide to a concentration of 0.1 M. The *p*-NP substrate was freshly diluted 15-fold with MilliQ-water to achieve a final concentration of 6.7 mM. A total of 25 µl of the citrate–phosphate buffer from the deepwell plate (see buffer) and 20 µl of the 6.7 mM substrate solution were added into a 96-well PCR plate. Finally, 25 µl of enzyme dilution was added and the solution was mixed. The plate was then sealed and incubated as described for the DNSA assay, but only incubated for 15 min to minimize the effects of instability of the *p*–NP substrates. After transferring the plate into ice water for cooling, 140 µl of 1 M Na_2_CO_3_ was added and mixed thoroughly to stop the reaction and allow detection of *p*-NP. Then, 100 µl was transferred into a 96-well F-bottom plate and the absorption at 405 nm (A_405_) was determined as mentioned above.

#### Glucose assay

The straw-based substrate (see enzymes and substrates section) was dispersed into water to a concentration of 2% (w/v) while constantly stirring. A total of 33.5 µl of buffer from the deepwell plate (see buffer section) and 16.5 µl of enzyme dilution were added to a PCR plate. Then, 50 µl of substrate solution was added to the PCR plate and the solution was mixed thoroughly. Since the straw-based substrate is insoluble, it was kept under continuous stirring for the duration of the pipetting to avoid sedimentation. The plate was sealed and incubated using the procedure described for the DNSA assay. After transferring the plate to ice water, the cooled plate was centrifuged at 1600×*g* for 5 min at 4 °C. The d-glucose HK assay from Megazyme was used to detect the released glucose. Solutions R1 and R2 were prepared as described in the manufacturer’s manual. A total of 25 µl of solution R2 and 10 µl of supernatant from the enzyme reaction were added to a new 96-well F-bottom plate. Finally, 200 µl of solution R1 was added and mixed thoroughly. The plate was then incubated at 37 °C for 30 min and the absorption at 340 nm (A_340_) was determined as mentioned above.

#### Contour plot

The raw data from the microplate reader were transferred into an Excel sheet (Microsoft, Washington, USA). The Excel sheet is supplied with this article (see Additional file 1) and can be used as a template, where all the necessary calculation steps are implemented as described below to convert the data into a SigmaPlot-compatible form. The first step is subtraction of the respective substrate blank value, as obtained from the tests described in the substrates section. Following this step, all values below zero are set to zero, because negative values would contort the color scheme of the contour plot. The data set is subsequently transformed into relative activity for each plate and the mean values among the three plates are determined for each well. The highest value from the average of all three plates is set to 100%. The data are then transformed into a SigmaPlot-compatible vertical XYZ triplet arrangement, which can be directly transferred into a SigmaPlot template (see Additional file 2). Furthermore, the standard deviation among the three plates is calculated for all 96 reactions. The contour plots were created using SigmaPlot 11.0 from Systat (Erkrath, Germany). The graph type was set to “contour plot”, graph style to “filled contour plot”, and data format to “XYZ triplet”. Detailed graph settings can be obtained from the provided template file. In addition, a legend for the activity is provided in Additional file 3.

#### Conventional pH and temperature plot

Celluclast^®^ was incubated with BBG at pH 5.5 using the citrate–phosphate buffer for 90 min at various temperatures between 35 °C and 75 °C. Furthermore, Celluclast^®^ was incubated with BBG at 45 °C, 55 °C, and 65 °C for 90 min using the standard eight citrate–phosphate buffers described in the buffer section. The activities were determined using the DNSA assay, as described above. Graphs were produced using GraphPad Prism^®^ (San Diego, CA, USA).

## Results and discussion

Creation of a contour plot from direct experimental data requires the use of a feasible high-throughput method and a considerable number of samples. The assay, therefore, has to be suitable for use in a 96-well plate design. The general advantages of this miniaturization from single reaction tubes to 96-well plates have already been shown for the DNSA and other colorimetric assays [14]. All assays used in this study were suitable for the 96-well plate format. To produce a contour plot, the enzyme and substrate mixtures were incubated at eight different pH levels. Those eight pH levels were incubated at 12 different temperatures using a gradient PCR cycler, resulting in 96 unique reaction conditions. A plate reader was used to obtain the data. The results were transformed into relative activities with the highest activity on each plate being set to 100%. The measurement was performed in triplicate and the averaged values were subsequently transformed into a contour plot using SigmaPlot, as described in the materials and methods section. The activity (*z*-axis) is represented as color from purple to red instead of a normal axis to enhance the view of the contour plot.

### Buffer system

One prerequisite for reliable determination of the pH and temperature optima is a stable buffer system that is suitable regarding the enzyme activity and nearly resistant to a pH shift with increasing temperature. Alternatively, this effect could be considered in the contour plot. The influence of the temperature on pK_a_ of the buffers and thereby pH is a fact that should be considered, especially for alkaline buffers such as Tris [15]. As a requirement for the assay, all pH variations of our citrate–phosphate buffer system were tested for change in pH between 35 °C and 80 °C (see Additional file 4). All buffers show a slight decrease in pH with rising temperature, with the buffers at higher pH values showing higher deviations. The temperature dependency increases with the amount of phosphate, which correlates with the temperature coefficients of phosphate (− 0.0028) and citrate (0). Since the temperature-dependent pH variations of all buffers were only minor, they could be neglected in the compilation of the contour plots within the range of values used here. Nonetheless, if another buffer system with a higher temperature coefficient should be utilized, it is easily possible to adapt the contour plot for the change in pH by assigning each buffer an individual pH at each temperature. These values are either experimentally determined as described in the respective materials and method section or derived from the temperature coefficients and can then accordingly be used to adapt the *x*-axis (pH) of the provided Sigmaplot file.

### Activity range determination for Cel8A

An overview of the different enzymes, substrates, and assays used in this study for the creation of the respective contour plots is given in Table 1.

**Table 1:** Overview of the different enzymes, substrates, and assay conditions

Enzyme	Assay	Substrate	Substrate conc. [mg/ml]	Enzyme conc. [µg/ml]	Assay duration [min]	Temp. gradient [°C]	pH gradient
Cel8A	DNSA	BBG	3.3	0.5	90	60.1 ± 19.9	4.0–8.0
	Azo-CMC	Azo-CMC	3.3	5	90	60.1 ± 19.9	4.0–8.0
Celluclast^®^	DNSA	BBG	3.3	5	90	55.0 ± 19.9	4.0–8.0
		AX	3.3	200	90	55.0 ± 19.9	4.0–8.0
	*p*-NP	*p*-NP-β-d-gluco-pyranoside	1.9 mM	50	15	55.0 ± 19.9	4.0–8.0
	Glucose HK	Wheat straw	10	250	90	55.0 ± 19.9	4.0–8.0

**Fig. 1:**
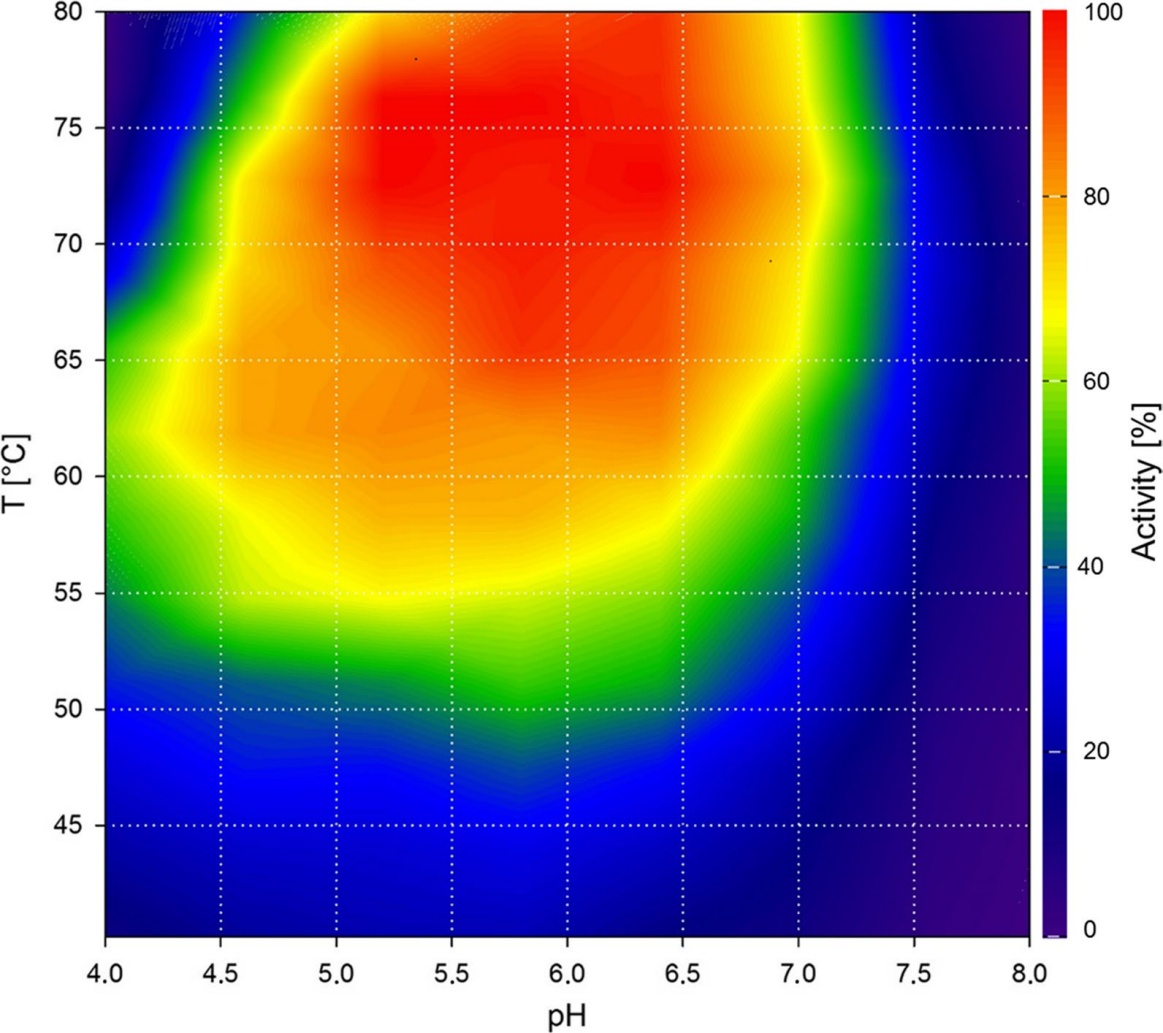
Contour plot of Cel8A using the DNSA assay with barley-β-glucan as substrate

Cel8A is a cellulase, more specifically an endo-glucanase, from *C. thermocellum* and was the first enzyme of glycoside hydrolase family 8 with a solved crystal structure [16]. In this study, the enzyme was incubated with BBG to validate our proposed method using the DNSA assay. Cel8A has been reported to have its temperature optimum at 75 °C and pH optimum between 5.5 and 6.5 [17]. These values are in accordance with the results of our contour plot as they are within the region of  > 90% activity (Fig. 1). Our contour plot, however, shows that the enzyme is highly active under a wide range of different conditions. The enzyme does exhibit acceptable activities at lower pH values when between 60 °C and 65 °C and also performs with more than 60% of its maximal activity when at pH values below 4.5. Our method provides the means of visualizing such effects and evaluating an enzyme’s performance at any point within the tested parameters at a glance.

**Fig. 2:**
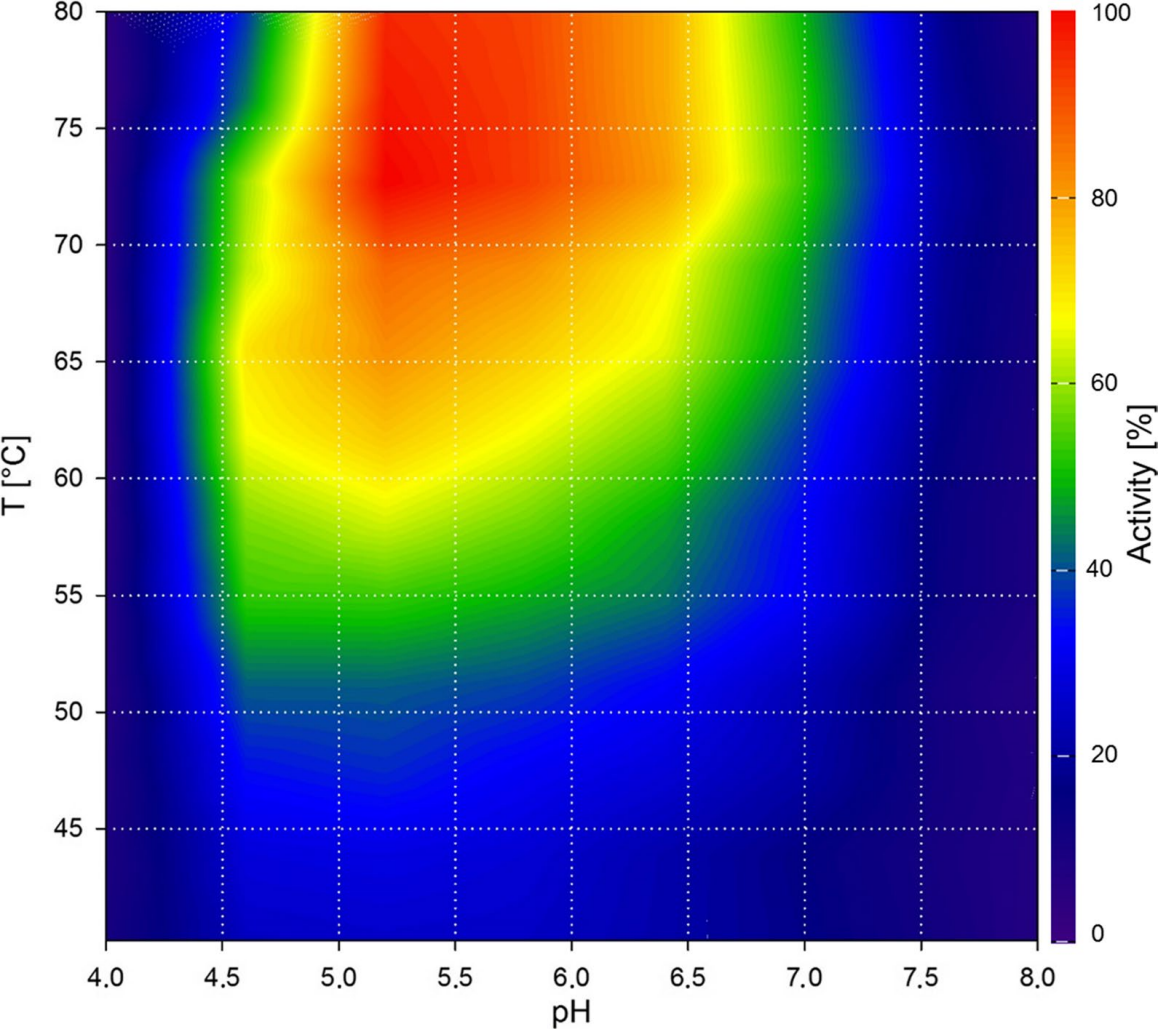
Contour plot of Cel8A using Azo-CM-cellulose

To test whether our method is suitable for different hydrolase assay methods, we produced a contour plot using Cel8A with Azo-CM-cellulose as substrate and the respective assay (Fig. 2). The plot shows similar optima at 75 °C and pH 5.5. However, the use of Azo-CM-cellulose as substrate resulted in a slightly smaller activity range. The minor differences between the two assays could be due to the dissimilar structures and linkage types of both substrates, thereby differing in their binding properties to the enzyme. Azo-CM-cellulose is a non-natural substrate with added dye groups, which could add further to this effect. Using Cel8A, we could show that our method provides valid data and is suitable for use in two different established assays for hydrolases: DNSA and Azo-CM-cellulose.

### Activity range determination for Celluclast^®^

**Fig. 3:**
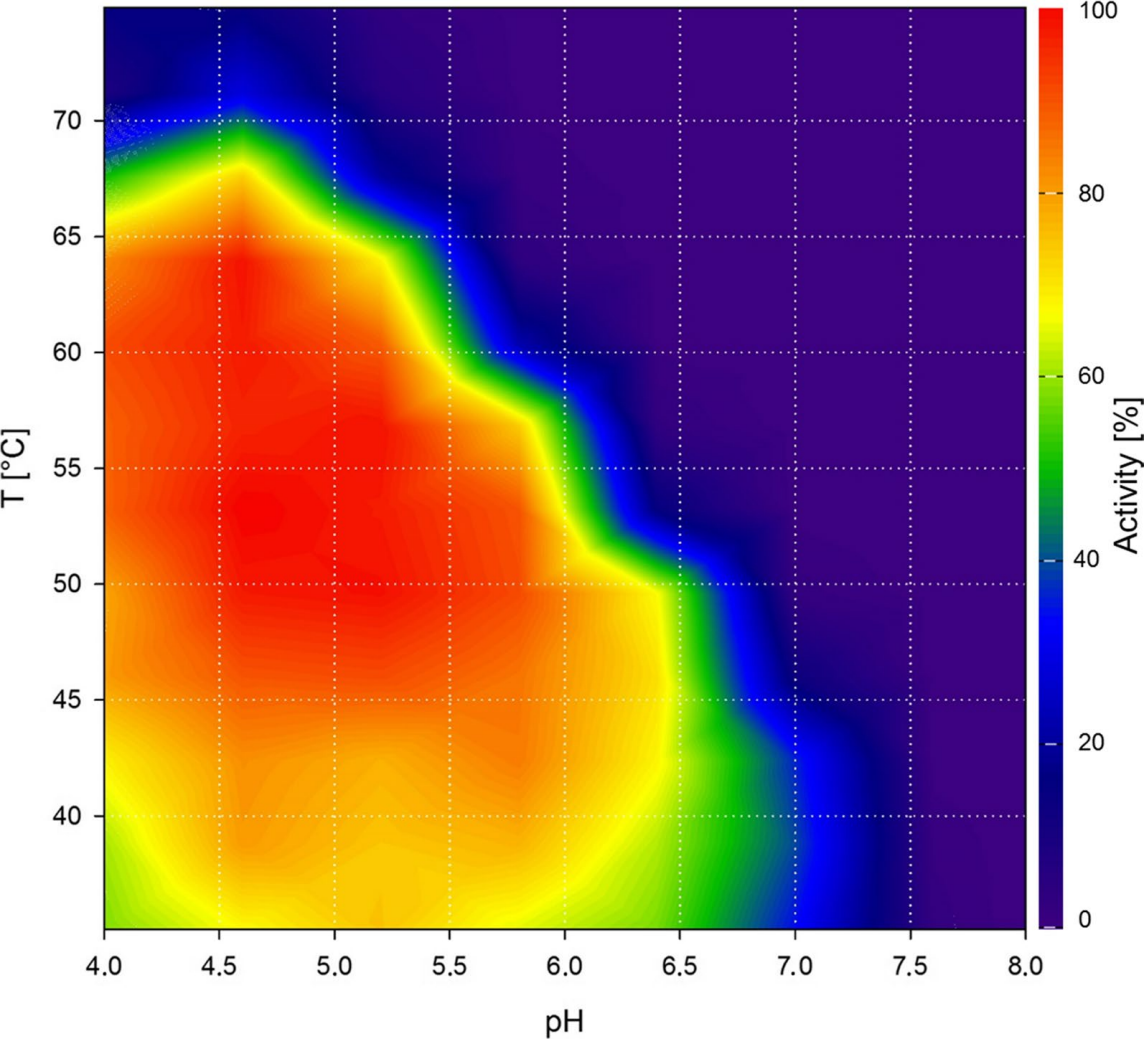
Contour plot of Celluclast^®^ using the DNSA assay with barley-β-glucan as substrate

**Fig. 4:**
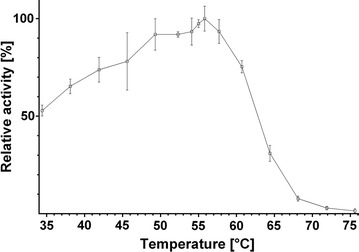
Conventional temperature optimum determination of Celluclast^®^ at pH 5.0. The temperature optimum of Celluclast^®^ on barley-β-glucan was determined at pH 5.0. The conventional approach shows the maximum activity around 55 °C and is in accordance with the results seen in the corresponding contour plot

**Fig. 5:**
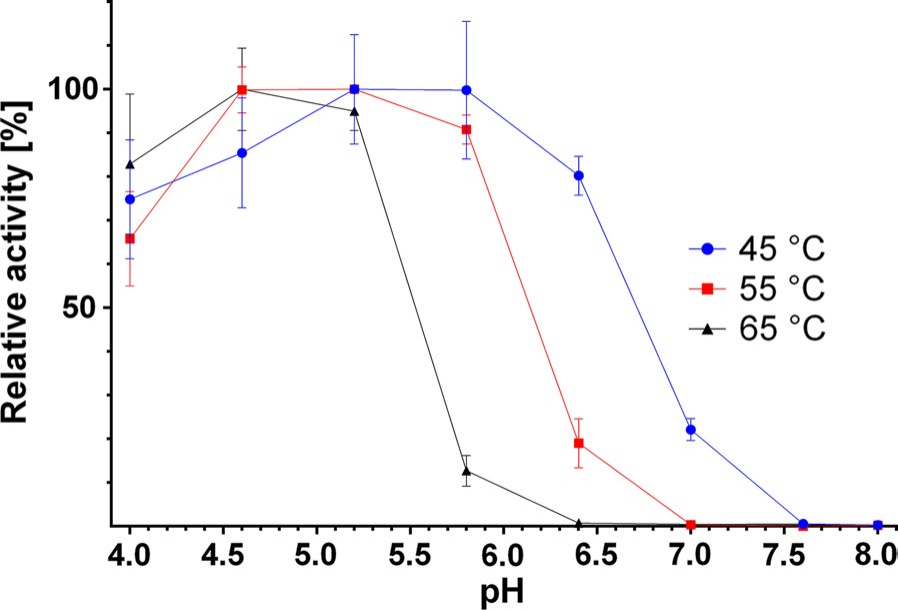
Conventional pH optimum determination of Celluclast^®^ at three temperatures. The pH optimum of Celluclast^®^ on barley-β-glucan was determined at 45 °C, 55 °C, and 65 °C. At lower temperatures, the enzyme can tolerate higher pH values. This is in accordance with the corresponding contour plot and shows the strong impact of the chosen fixed parameter when determining the pH or temperature optimum separately

In addition to a single enzyme, an enzyme mixture was studied. Celluclast^®^ is a widely used commercial cellulase product derived from *Trichoderma reesei*. It consists mostly of cellobiohydrolases and endo-1,4-β-glucanases, but also features xylanases and at least one β-xylosidase [18]. The product manual states that the optimal activities are between 50 °C and 60 °C and pH 4.5 and 6.0 [19]. In this work, we first produced a contour plot for Celluclast^®^ with BBG as a substrate using the DNSA method (Fig. 3). The BBG contour plot was verified by a standard temperature curve (Fig. 4). The results of the temperature curve at pH 5.5 are in accordance with the results of the contour plot, showing the same range of activity. However, the measurement of separate curves is inadequate to describe the activity of Celluclast^®^ on BBG. The range of pH in which the enzyme mixture shows high activity is strongly temperature dependent. The higher the temperature, the lower the pH has to be to achieve a high activity. Around 45 °C, Celluclast^®^ is highly active (> 60%) up to a pH of 6.5, whereas at 65 °C, it is highly active only up to a pH of 5.0. Describing the activity with two separate curves is, therefore, strictly dependent on the value chosen as the static parameter. Temperature graphs, for example, those taken at the two extremes of the stated pH range (4.5 and 6.0) should give significantly different results. The same holds true for pH graphs taken at different temperatures. To make this effect evident, we produced standard pH graphs at 45 °C, 55 °C, and 65 °C (Fig. 5). These three pH curves show that, with increasing temperature, the pH border for high activity shifts to more acidic pH values. The three curves thereby verify the results seen in the contour plot. Furthermore, they show the limitations of the conventional separate activity determination, which at least for Celluclast^®^ is, in the case of the pH curves, dependent on the chosen temperature. The use of our contour plot method circumvents this problem completely by measuring the effect of temperature and pH in one step.

**Fig. 6:**
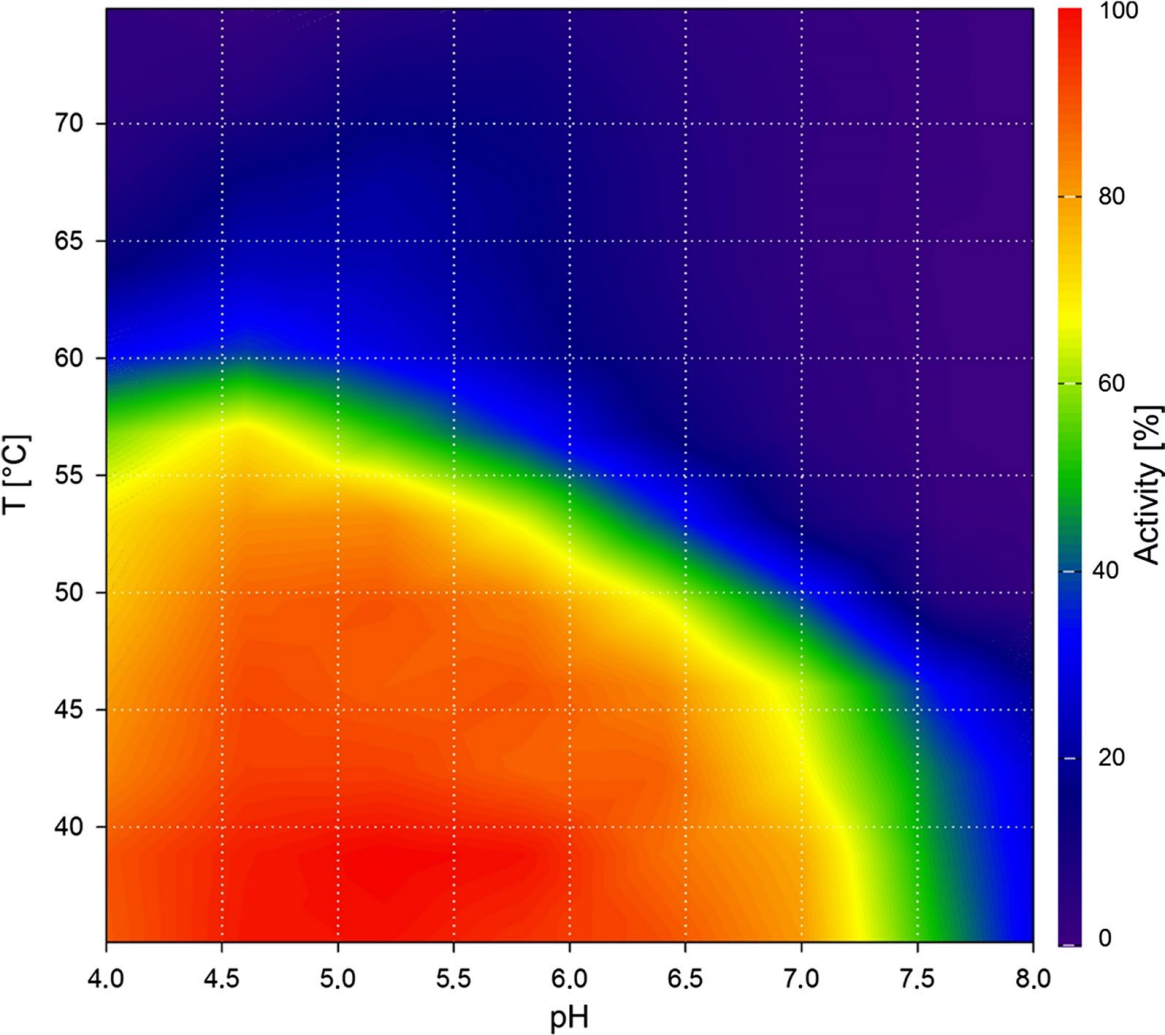
Contour plot of Celluclast^®^ using the DNSA assay with arabinoxylan as substrate

The activity range of Celluclast^®^ on arabinoxylan (AX) (Fig. 6) differs from that with BBG as a substrate. The activity is notably shifted toward lower temperatures, with no high activities observed above 60 °C. As Celluclast^®^ is a mixture of several enzymes, it is most probable that these different enzymes have different characteristics. However, the activity pattern is similar to that of BBG; for example, at pH 4.5, the enzyme mixture is highly active up to almost 60 °C, whereas at pH 6.5, it is only highly active up to 50 °C. In the literature, the optimum of Celluclast^®^ on wheat soluble AX was determined by a response surface model (RSM) and found to be around pH 4.4 and 39 °C [19]. Our contour plot shows the highest activity in the same temperature range and at a slightly higher pH, which is still within the optimum determined by the RSM. While the optimum is nearly the same for both methods, our contour plot strikingly shows the exact activity of Celluclast^®^ on AX within the entire range of the tested conditions and exhibits a more detailed disposition than the RSM. With our contour plots of Celluclast^®^ on BBG and AX, we could show that complex enzyme mixtures can be analyzed with the proposed method.

**Fig. 7:**
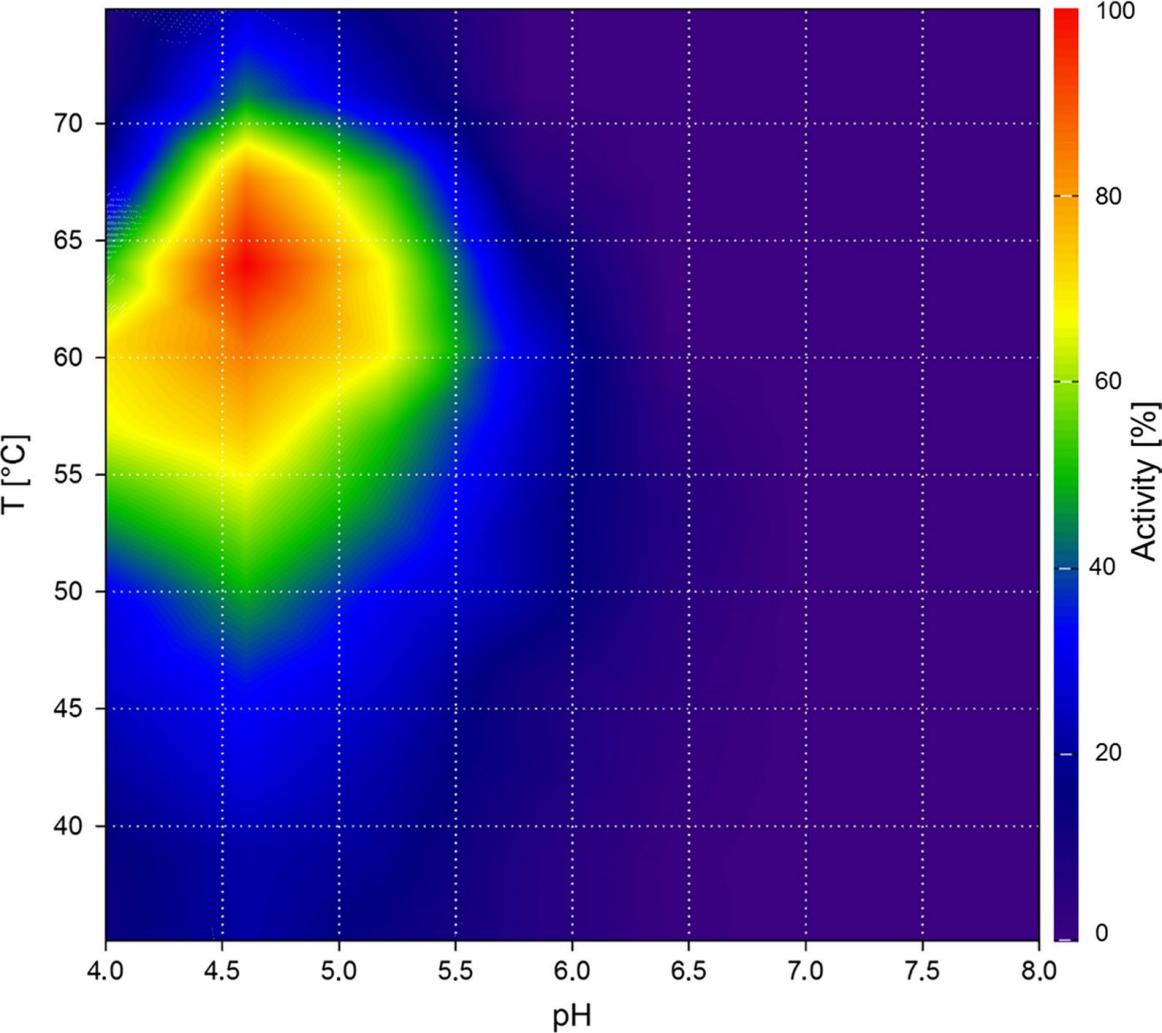
Contour plot of Celluclast^®^ using *p*-NP-β-d-glucopyranoside

To demonstrate further the versatility of our activity range determination, we evaluated its use with additional substrates and assays. The activity of Celluclast^®^ on *p*-NP-β-d-glucopyranoside was tested and the contour plot is shown in Fig. 7. High activity on this substrate is limited to a rather narrow range between pH 4.0 to 5.5 and 55 °C to 70 °C. This suggests that only one or a few enzymes within the Celluclast^®^ enzyme mixture probably shows activity toward *p*-NP-β-d-glucopyranoside. *p*-NP glycosides can be used as substrates for the preparation of contour plots. However, not all *p*-NP glycosides are suitable substrates in the assay, as several of them show high background due to instability (see Additional file 5), especially when high temperatures are combined with high pH values.

**Fig. 8:**
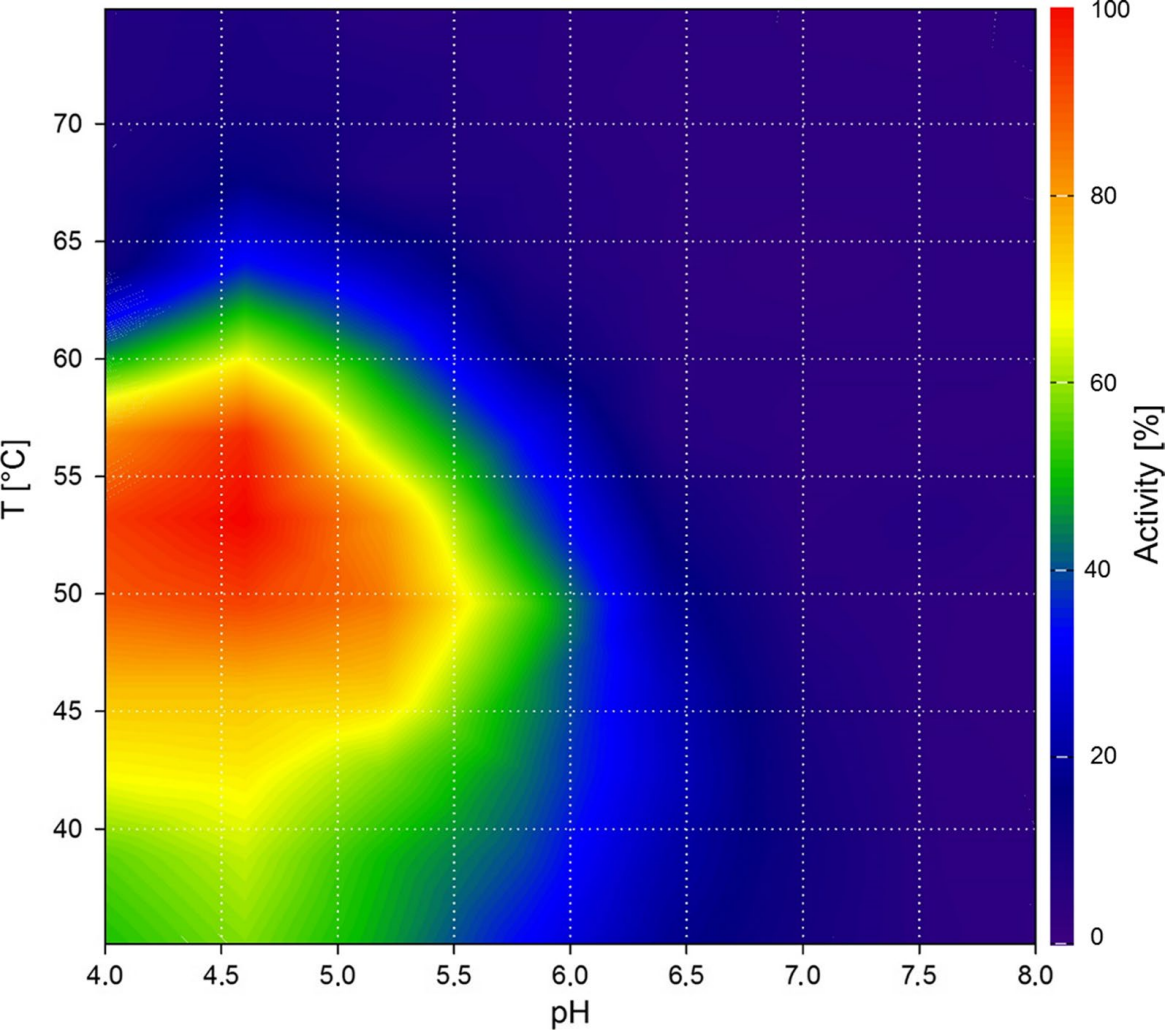
Contour plot of Celluclast^®^ using the d-glucose HK assay with a straw-based natural substrate

To test the application of our method on a natural biomass sample, we chose to use a straw-based substrate. Liberation of glucose by Celluclast^®^ was detected using the commercial d-glucose HK assay (Megazyme). The main component in the cell wall of straw is cellulose [20], which is the major source of enzymatically liberated glucose. The contour plot (Fig. 8), therefore, predominantly shows the activity of Celluclast^®^ on crystalline cellulose, which is recalcitrant toward enzymatic degradation [21]. The highest release of glucose was observed at approximately pH 4.0 to 5.5 and 40 °C to 60 °C. The range with high activity is, therefore, less wide than determined for BBG, which should be taken into account for a process mainly aimed to degrade cellulose. With the use of d-glucose HK and *p*-NP-β-d-glucopyranoside as alternative assays, we could demonstrate the adaptability of our method to a total of four different and commonly used glycoside hydrolase assays. The usage of straw showed that our method is also suitable for natural substrates of industrial relevance and might be used for the evaluation of complex processes and substrates. Celluclast^®^ shows varying activity profiles on different substrates. Collectively for all methods, it is, therefore, crucial to determine the pH and temperature range data of an enzyme with the substrate of interest.

### Considerations when using the method

When a contour plot is produced with the proposed method, there are several considerations that should be taken into account. The substrate has to be stable and the measured background reproducible over the complete range of conditions in the 96-well plate. This is a prerequisite, because the substrate control has to be subtracted from all values prior to the conversion into relative activities, but cannot be directly measured on the plate for each of the 96 conditions. Several *p*-NP glycoside substrates, for example, cannot be used, since they show a highly temperature- and pH-dependent background. Very precise pipetting is necessary to yield acceptable levels of standard deviation from the triplicates. The use of 96-head and 8-channel pipettes is strongly recommended as they significantly improve the accuracy and accelerate the procedure. Moreover, plate reader and gradient PCR cycler are technical requirements to execute the method properly. The temperature range in which the method can be performed can be adapted in accordance with the technical properties of the gradient PCR cycler, which is usually limited to 30 °C or 40 °C and often cannot include values below 20 °C. We directly converted the absorbance into relative activity of an enzyme, because the values are all on a linear calibration curve (data not shown). If that is not the case, however, the activity has to be calculated first, for example, in U/mg, and these values can then be used to produce the contour plot. When wanting to produce a contour plot for an enzyme that is strongly dependent on divalent metal ions, a different buffer system has to be used, since citrate–phosphate-based buffers complex those ions.

While RSM approaches are an indisputably powerful tool to assess complex relationships, for example, diverse variables affecting multiple factors, determining the effect of only pH and temperature on the activity is not too complex to be achieved by direct measurement. Determination of the correct border values of the model can take several approaches and may influence the resulting model. The RSM model is derived from only a small number of measurements around the central point of the enzyme’s activity and the accuracy toward the actual experimental values has to be tested afterwards. The overall resolution of the RSM is, therefore, lower than obtained with our method, which makes it difficult to see activity effects as shown for Celluclast^®^ at higher temperatures and/or pH values. Our method does not require the ability to design complex statistical models and can be performed with minimal and relatively standard technical requirements. A drawback both our method and RSM share is that direct visualization of the standard deviation within the three-dimensional plot is not possible. However, our method allows calculation of the standard deviation for each individual data point. Most RSM models determine the standard deviation for the whole model only from data of the central data point of the model, while the other data points are only measured once [8, 10, 22]. The standard deviations are naturally higher at the borders of an enzyme’s activity and illustrate a strong impact on the activity within a slight change of conditions. Around the optimum and within areas of no or low activity, deviations are significantly lower. Since the proposed method results in a full factorial data set, it would be possible to calculate a statistical model if needed for further analysis. The derived statistical model would base directly on experimental data, but is an additional step which should not be necessary for standard applications of the method.

In summary, easy assessment of an enzyme’s or an enzyme mixture’s suitability for a combination of process parameters is of crucial importance when designing biotechnological processes on either laboratory or industrial scale. Temperature and pH are two of the most important process factors influencing enzyme activity. Conventional approaches to test the effect of those factors on enzymes are based on the assumption that there is a two-dimensional correlation between temperature and activity as well as pH and activity. In contrast, new approaches determine the relationship in three-dimensional matter, but are statistics-based, complex to design and their accuracy to the actual data may vary. The method introduced here on the other hand allows fast and easy determination of the effect that the temperature and pH have on the enzyme’s activity simultaneously using 96-well plates, multichannel pipettes, and a gradient PCR cycler. With this basic technical equipment, it is possible to produce contour plots of pH, temperature, and activity that are based directly on experimental data. The method was tested for several widespread glycoside hydrolase assays as well as model and complex substrates.

## Conclusions

The proposed method offers a new approach to evaluate enzymes for their use in lab-scale experiments as well as biotechnological applications in regard to their pH and temperature characteristics. The use of a 96-well format and a gradient PCR cycler allowed the simultaneous determination of pH and temperature optima. Testing has confirmed that the method is suitable for enzymes as well as enzyme mixtures and different assays. The experimental procedure is fast and the obtained data can be converted into a contour plot with only a few very simple steps. Since the contour plot is directly derived from a large number of independent data points, it does not require further verification. The method showed several advantages over existing methods and is a supreme tool to evaluate an enzyme’s activity profile in regard to pH and temperature. While only demonstrated for glycoside hydrolases, the method should be applicable to other kinds of enzymes such as proteases as well. A general application of the method when characterizing single enzymes or enzyme mixtures would greatly enhance the information on the relationship among pH, temperature, and activity.

## Additional files

**Additional file 1:** Contour plot data transformation template.

**Additional file 2:** SigmaPlot template.

**Additional file 3:** Activity scale for contour plots.

**Additional file 4:** pH variations of citrate–phosphate buffer system dependent on temperature.

**Additional file 5:** Determination of pNP-substrate stability.

## Abbreviations

AX: arabinoxylan
BBG: barley-β-glucan
CM-cellulose: carboxymethyl-cellulose
DNSA: 3,5-dinitrosalicylic acid
HK: hexokinase
*p*-NP: *para*-nitrophenol
RSM: response surface model
