# Supplementary figures and images for: A new method to evaluate temperature vs. pH activity profiles for biotechnological relevant enzymes
Source: Biotechnol Biofuels. 2017 Oct 11;10:234. doi: 10.1186/s13068-017-0923-9 (PMC5637330; doi:10.1186/s13068-017-0923-9)

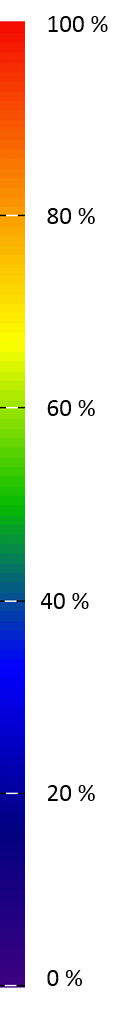

Supplement: Supplementary file 3 — Additional file 3: Activity scale for contour plots. [file 13068_2017_923_MOESM3_ESM.png]

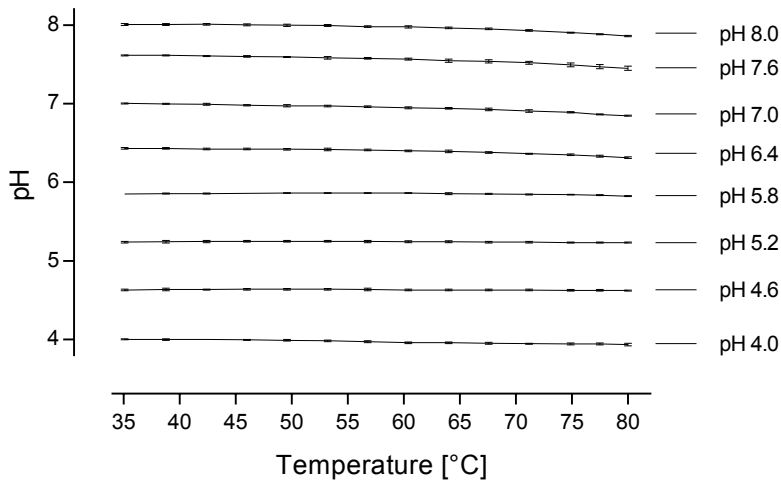

Supplement: Supplementary file 4 — Additional file 4: pH variations of citrate–phosphate buffer system dependent on temperature. [file 13068_2017_923_MOESM4_ESM.pdf]
