# Supplementary material for: A new method to evaluate temperature vs. pH activity profiles for biotechnological relevant enzymes
Source: Biotechnol Biofuels. 2017 Oct 11;10:234. doi: 10.1186/s13068-017-0923-9 (PMC5637330; doi:10.1186/s13068-017-0923-9)
Supplement: Supplementary file 3 [file 13068_2017_923_MOESM5_ESM.docx]

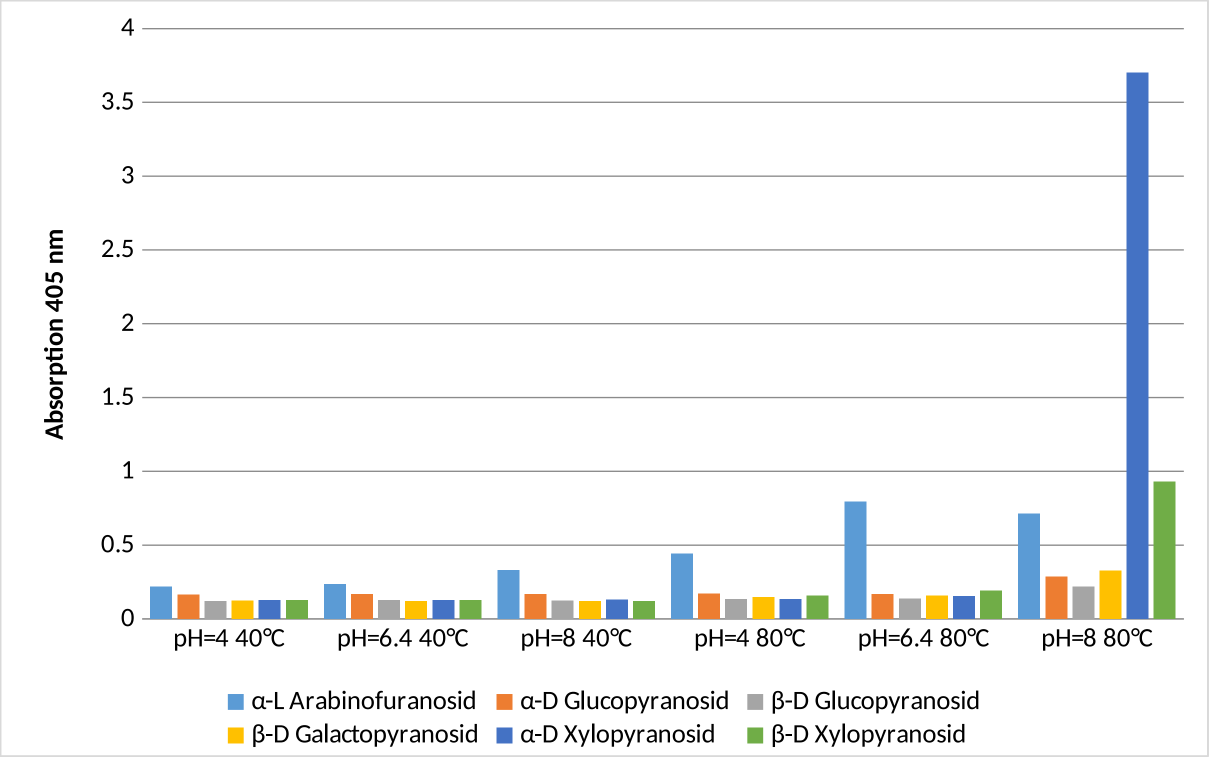

Absorption 405 nm
4
3.5
3
2.5
2
1.5
1
0.5
0
pH=4 40℃
pH=6.4 40℃
pH=8 40℃
pH=4 80℃
pH=6.4 80℃
pH=8 80℃
α-L Arabinofuranosid
α-D Glucopyranosid
β-D Glucopyranosid
β-D Galactopyranosid
α-D Xylopyranosid
β-D Xylopyranosid
